# Cytokine Signature Unveils Subgroups of Patients With Immune‐Mediated Sensory Neuronopathies

**DOI:** 10.1111/jns.70008

**Published:** 2025-03-04

**Authors:** Christian P. Moritz, Yannick Tholance, Coralie Borowczyk, Fabienne Jospin, Stéphane Paul, Jean‐Christophe Antoine, Jean‐Philippe Camdessanché

**Affiliations:** ^1^ Synaptopathies and Autoantibodies (SynatAc) Team, Institute MeLis NeuroMyoGène, INSERM U1314/CNRS UMR 5284 Université Claude Bernard Lyon 1 Lyon France; ^2^ University Jean Monnet Saint‐Étienne France; ^3^ Department of Neurology University Hospital of Saint‐Etienne Saint‐Etienne France; ^4^ Department of Biochemistry University Hospital of Saint‐Etienne Saint‐Etienne France; ^5^ CIRI – Centre International de Recherche en Infectiologie, Team GIMAP, Univ Lyon, Université Claude Bernard Lyon 1, Inserm, U1111, CNRS, UMR530, CIC 1408 Vaccinology Saint‐Etienne France; ^6^ Department of Immunology and Biotherapies University Hospital of Saint‐Etienne Saint‐Etienne France

**Keywords:** anti‐cytokine antibodies, cytokine signature, cytokines, interleukins, sensory neuronopathy

## Abstract

**Background and Aims:**

Immune‐mediated sensory neuronopathies (SNN) can occur alongside autoimmune disorders (e.g., Sjögren syndrome), involve autoantibodies (such as anti‐FGFR3 or anti‐AGO antibodies), or present in isolation. The underlying mechanisms remain unclear. This study aimed to investigate the role of proinflammatory cytokines in these conditions.

**Methods:**

Blood levels of IL‐1β, IL‐6, IL‐17, TNF‐α, INF α‐2, and INF‐γ were measured using a Bioplex T200 platform and the Bio‐Plex Pro Reagent Kit III in 113 patients with SNN between 2.4 and 464.4 months after symptom onset, categorized based on disease course (acute, subacute, chronic). Eighteen patients had anti‐AGO antibodies, 48 had anti‐FGFR3 antibodies, and 14 had an autoimmune disease without detectable anti‐AGO or FGFR3 antibodies. Disease extent was measured by the SNN score, while the disease severity was evaluated using the modified Rankin score. Immunoreactivity against IL‐6 and INF‐γ was measured via ELISA.

**Results:**

Multicomponent analysis utilizing cytokines levels identified four distinct patient subgroups characterized by differences in age at onset and SNN score. No significant differences were observed among the subgroups regarding disease course and severity, presence of anti‐AGO or anti‐FGFR3 antibodies, or association with an autoimmune disease. A small subgroup of three younger patients exhibited the highest levels of TNF‐α, IL‐6, and IL‐1β. Another subgroup of seven patients displayed elevated INF α‐2 levels and tended towards highest SNN scores. The largest group (95 subjects) comprised older individuals with relatively lower cytokine levels and decreased anti‐IL‐6 immunoreactivity.

**Interpretation:**

These cytokine profiles suggest diverse underlying mechanisms within immune‐mediated SNN. Further investigation is warranted to determine whether certain profiles, particularly those involving young patients with elevated proinflammatory cytokines, might benefit from targeted treatments.

## Graphical abstract

We investigated cytokine levels in 113 patients with immune‐mediated sensory neuronopathies (SNN) with varied disease manifestations. We identified 4 patient subgroups based on cytokine profiles. One small subgroup had high TNF‐α, IL‐6, and IL‐1β and was younger. Findings suggest different SNN mechanisms, which might be a potential for targeted treatments.

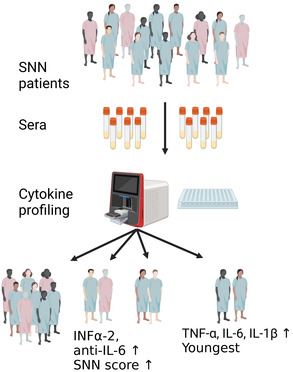

## 1 Introduction

Sensory neuronopathies (SNN), also known as sensory ganglionopathies, are characterized by the selective targeting of sensory neurons located within the dorsal root ganglia (DRG). Regardless of their etiology, a common thread linking various forms of SNN is the involvement of the immune system. A distinctive immunopathological profile has emerged, marked by lymphocyte infiltration into the DRG, primarily consisting of cytotoxic T‐cells. This cellular invasion coincides with the upregulation of major histocompatibility complex (MHC) molecules on both neurons and their surrounding satellite cells, ultimately leading to neuronal damage [1].

Initially described in paraneoplastic SNN [2], this immune‐driven pathology is also observed in SNN linked to Sjögren syndrome [3] and in certain idiopathic cases lacking a clear underlying cause [4]. This finding strongly suggests a crucial role for cell‐mediated immunity in SNN pathogenesis. Moreover, evidence indicates a contribution from humoral immunity, demonstrated by the presence of B‐cells, immunoglobulin G (IgG) deposition within the DRG, and circulating antibodies. These antibodies have been detected in paraneoplastic SNN, where they target antigens such as Hu and CRMP5 [5]. In non‐paraneoplastic variants, antibodies directed against fibroblast growth factor receptor 3 (FGFR3) [6] or Argonaute (AGO) [7] has also been reported, though further investigation is needed to clarify their significance [8].

In addition to antibodies, cytokines are key players in the immune response, participating in both innate and adaptive immunity. They play a pivotal role in the pathogenesis of autoimmune diseases. However, the precise triggers for the breakdown of self‐tolerance and the subsequent events leading to the induction of pathogenic autoimmune responses remain largely undefined for most autoimmune diseases. Studies in experimental models of human autoimmune diseases and clinical observations in patients have revealed a general pattern: proinflammatory cytokines contribute to the initiation and perpetuation of autoimmune inflammation, while anti‐inflammatory cytokines promote the resolution of inflammation and recovery from the acute phase of the disease [9, 10, 11, 12, 13]. Anti‐cytokine autoantibodies (ACAAs) are increasingly recognized as modulators of disease severity in infection, inflammation, and autoimmunity [14]. By reducing or augmenting cytokine signaling pathways or by altering the half‐life of cytokines in circulation, ACAAs can exert either pathogenic or disease‐ameliorating effects [14, 15, 16]. The origins of ACAAs remain unclear, as does the role of cytokines in SNN.

We present a study investigating six circulating proinflammatory cytokines and two corresponding ACAAs in a cohort of patients with non‐paraneoplastic SNN. Our findings demonstrate that the cytokine profile can differentiate subgroups of patients, suggesting varying underlying mechanisms driving the inflammatory response in immune‐mediated SNN.

## 2 Material and Methods

### 2.1 Standard Protocol Approvals, Registrations, and Patient Consents

The retrospective case/control and observational study involves the use of sera from human subjects. It was approved by the ethics committee of the University Hospital of Saint‐Etienne, France (IRBN742021/CHUSTE), and has been carried out in accordance with the Code of Ethics of the World Medical Association (Declaration of Helsinki). The privacy rights of human subjects were maintained. No animal experiments were conducted for this study.

### 2.2 Patients

One hundred and thirteen patients with a diagnosis of SNN according to published criteria entered in the study [17]. The following information were collected: age at onset of the neuropathy, neuropathy onset (acute: < 1 month; subacute: > 1 month < 6 months; chronic: > 6 months), delay between onset and serum collection, presence of anti‐FGFR3 or AGO antibodies according to previously published ELISA methods [18], or of an identified associated disease such as Sjögren syndrome or systemic lupus erythematosus (SLE), immunomodulatory treatment, improvement under treatment as reported by the physician in charge of the patient, initial modified Rankin score (mRS), and if available post treatment mRS. The extension of the SNN was measured by the SNN score [17]. By definition, all the patients had a SNN score > 6.5. Patients with a SNN score > 10 were considered as those having the most extensive disease since this implies involvement of the four limbs and a severe reduction of sensory action potentials (SAP) in the upper limbs. Of note, the SNN score is not a measure of disability since it is not correlated with the mRS (data not shown). To define seropositivity of anti‐cytokine antibodies, we tested sera of 77 healthy donors.

Serum samples were obtained in the morning at the patient's visit mostly at time of diagnosis and prior to any immunomodulatory treatment. Samples were then frozen and stored at −80°C until utilization.

### 2.3 Measure of Cytokine

Serum levels of six cytokines were measured using a Bioplex T200 platform, Bio‐Plex ProTM Reagent Kit III and the following BioRad cytokine kits according to the instructions of the manufacturer: interferon (INF) α‐2 (171B6010M), IFN‐γ (171B5019M) interleukin (IL)‐1 β (171B5001M), IL‐6 (171B5006M), IL‐17 (171B5014M), and tumor necrosis factor (TNF)‐α (171B5026M).

### 2.4 Detection of Anti‐IL‐6 and IFN‐γ Immunoreactivity

An ELISA assay has been developed to detect anti‐IL6 and INF‐γ autoantibodies using our established protocol [19] including the control for serum‐specific background noise [18]. Minor modifications were the following: we coated ELISA plates with recombinant human IL6 (0.3 μg/mL, Sino Biological, reference 10 395‐HNAE) or human IFN‐γ (2 μg/mL, Sino Biological, reference 11 725‐HNAE) in 0.1 M carbonate/bicarbonate, pH 9.6, without glycerol. Sera were diluted 1:300 and secondary antibody 1:2000. Signals were revealed with 1‐StepTM Ultra TMB‐ELISA substrate (Thermo scientific, reference 34 029) for 20 min and the reaction stopped with 2 M sulfuric acid. The *reactivity* against these proteins served as an indicator of the *concentration* of these autoantibodies. Hence, the latter term will be used in the rest of the manuscript.

Positivity of the anti‐cytokine antibody was defined by a cut‐off of 4 standard deviations above the immunoreactivity of 77 (or 63 for IFN‐γ) healthy controls.

### 2.5 Statistical Methods

Statistical analyses were performed using IBM SPSS 20 statistics software. Comparison of qualitative variables used the Chi^2^ test or the exact Fisher's test when appropriate. According to their distribution, comparative variables were compared by the ANOVA test and the T tests, or the Mann–Whitney and the Kruskal Wallis tests. Principle component analysis (PCA) was performed without rotation after exclusion of incomplete data. Rho Spearman test including significance test (two‐sided) was used for correlation analysis.

## 3 Results

Our cohort consisted of 113 patients, including 47 males and 66 females, with ages ranging from 19 to 89 years (mean age 48.4 ± 15.9 years). Neuropathy onset was classified as acute in 13 patients, subacute in 24 patients, and chronic in the remaining 76 patients. Biological samples were collected between 2.4 and 464.4 months after disease onset (median 69.6 months). Median delay between onset and sample collection was 16.8 months for acute forms, 62.4 months for subacute forms, and 77.4 months for chronic forms.

Among the participants, 18 patients were previously tested positive for anti‐AGO antibodies, 48 for anti‐FGFR3 antibodies, and 14 had an associated autoimmune disease without detectable anti‐AGO or FGFR3 antibodies. The remaining 33 patients showed no indication of an underlying autoimmune condition. Initial mRS scores ranged from 0 to 5, with a mean of 2.6 ± 1.2. An SNN score greater than 10 was observed in 78 cases. Treatment response data, primarily concerning intravenous immunoglobulin (IVIg) therapy, was available for 47 patients.

### 3.1 Systematic Cytokine Profiling Identifies Subgroups in Immune‐Mediated SNN

**FIGURE 1:**
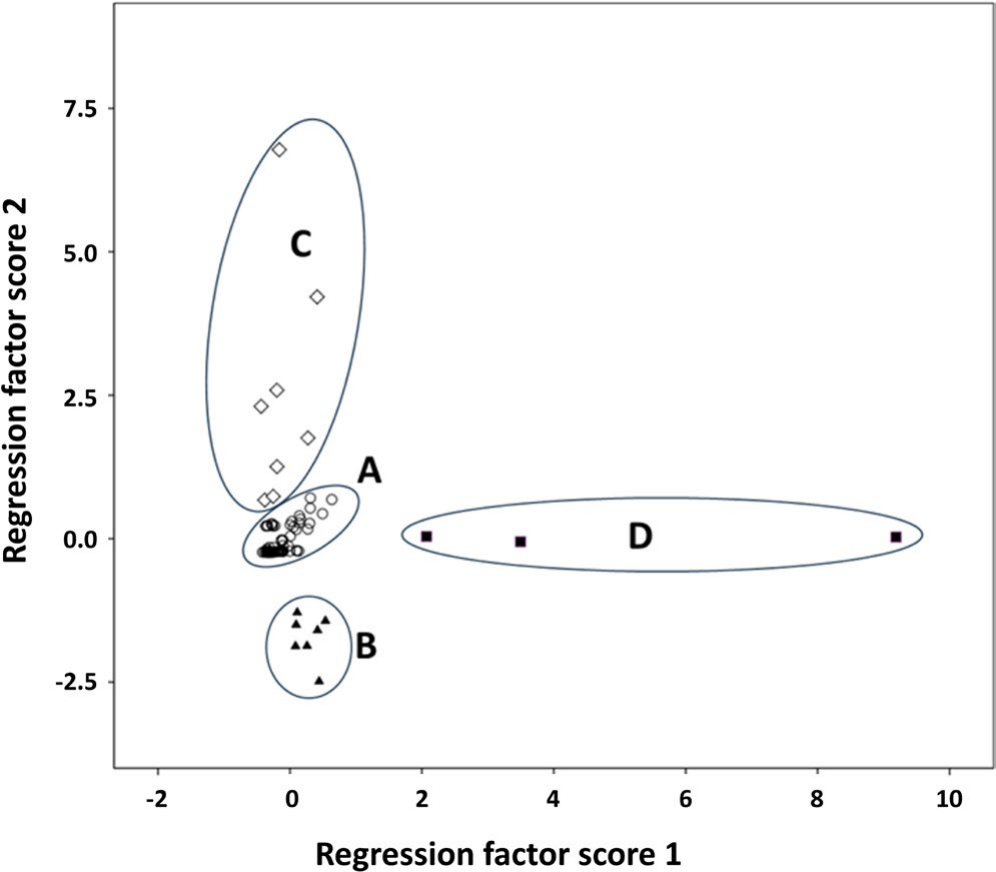
Identification of four SNN patient subgroups through principle component analysis (PCA) of cytokine levels. PCA performed on the serum levels of six cytokines in 113 SNN patients reveals four distinct patient subgroups (labeled A–D).

PCA was performed using blood concentrations of IL‐1β, IL‐6, IL‐17, TNF‐α, INF α‐2, and INF‐γ. This generated a two‐dimensional space representing 66% of the total variance, visualized through the first two regression factor scores (Figure 1). Visual inspection of the resulting plot before any statistical analysis revealed four discernible patient clusters. Groups B and D were distinctly separated, while two patients from group C fell near group A. Given that group A was the largest, these borderline cases were assigned to group C to minimize potential information loss within this small group.

**FIGURE 2:**
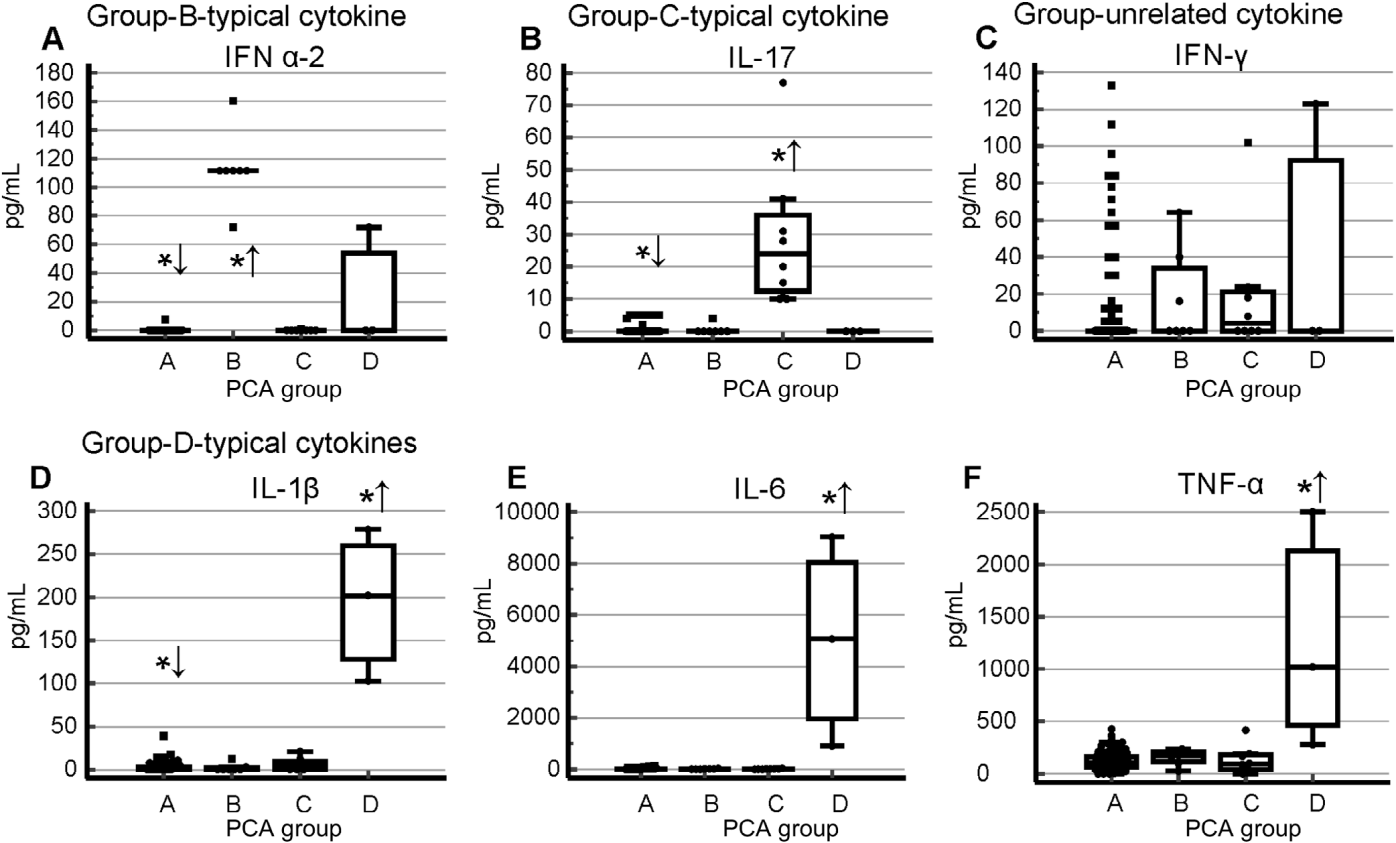
Distribution of cytokine levels across four SNN subgroups. The symbols *↑ and *↓ indicate statistically significant differences (higher and lower, respectively) in mean cytokine levels compared to the combined dataset of the remaining groups (*p* ≤ 0.05, Mann–Whitney test).

**FIGURE 3:**
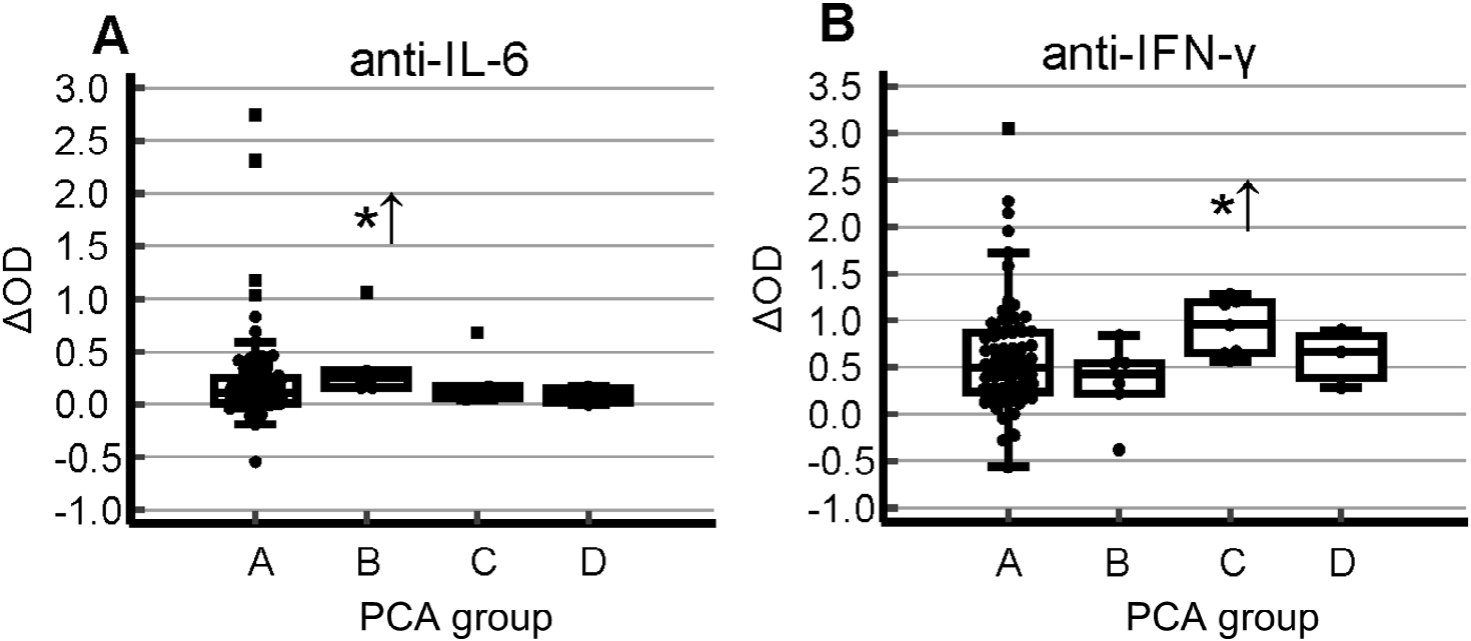
Distribution of anti‐cytokine immunoreactivities across four SNN subgroups. The symbol *↑ indicates significantly higher mean levels compared with the combined dataset of the remaining groups (*p* ≤ 0.05, Mann–Whitney test).

Figures 2 and 3 display the cytokine levels for all six cytokines and the corresponding anti‐cytokine immunoreactivities for two of them, respectively. Table 1 summarizes the characteristics of the four identified SNN subgroups. Notably, the mean delay between disease onset and blood sampling for serum analysis did not vary significantly among the four groups (Table 1).

**TABLE 1:** Characteristics of the four identified SNN subgroups.

Subgroup	A	B	C	D
Number	95	7	8	3
Age at onset [years]	50.4 ± 14.6**	32.8 ± 11.9**	47.1 ± 24.4	28.0 ± 2.9**
Male [*n*]	40	2	2	2
SNN score	11.0 ± 1.7	12.1 ± 1.1*	10.0 ± 2.3	11.5 ± 2.1
SNN score > 10 [*n*]	27	7*	3*	1
Onset chronique [*n*]	49	6	4	1
Delay [months]	79.2 ± 114.8	112.1 ± 61.9	128.9 ± 165.5	76.8 ± 66.7
Anti‐FGFR3 Ab [*n*]	42	1	4	1
Anti‐AGO Ab [*n*]	16	1	1	0
Isolated AID [*n*]	11	2	1	0
mRankin score	2.6 ± 1.3	3.0 ± 1.0	2.7 ± 1.0	4.0 ± 0.9
Response to treatment [*n*/total]	9/40	1/3	0/3	—
TNF‐α [pg/mL]	123.0 ± 81.2*	158.7 ± 73.2	131.0 ± 131.7	1265.7 + 1130.7**
IFN α‐2 [pg/mL]	0.08 ± 0.8**	112.7 ± 25.7**	0.1 ± 0.3	23.9 ± 41.5
IFN‐γ [pg/mL]	11.4 ± 27.6*	17.1 ± 25.5	19.0 ± 34.8	41.0 ± 71.0
IL‐1β [pg/mL]	2.5 ± 5.3**	2.1 ± 4.5	5.9 ± 8.0	194.7 ± 88.2**
IL‐6 [pg/mL]	14.0 ± 21.3	10.4 ± 9.8	10.5 ± 7.4	5002.0 ± 4057.4**
IL‐17 [pg/mL]	0.66 ± 1.7**	0.6 ± 1.5	29.0 ± 1.7**	0.00 ± 0.00
Anti‐IL‐6 Ab [ΔOD]	0.22 ± 0.46	0.37 ± 0.35**	0.19 ± 0.22	0.08 ± 0.08
Anti‐IFN‐γ Ab [ΔOD]	0.60 ± 0.58	0.35 ± 0.41	0.92 ± 0.29**	0.61 ± 0.55

*Note:* The statistical analysis was performed by comparing each group with the combined dataset of the remaining groups. **p < 0.05; *p > 0.05 and < 0.10 when comparing a given group to the other groups together. Anti‐cytokines antibody levels are measured in optic density. Ab: antibody; AID: autoimmune disease; *n*: number; ΔOD: difference of optical densities between reactivity against cytokine and uncoated well.

Group A constitutes the majority of patients (95 cases; Figure 1). Compared to the combined dataset of the other groups, these patients exhibited significantly lower circulating levels of IL‐1β, IL‐17, and IFN α‐2 (Figure 2, Table 1). TNF‐α and IFN‐γ were also marginally lower in this group. Clinically, group A was notable for a significantly older age.

Group B comprised a homogenous subset of 7 patients, characterized by significantly elevated IFN α‐2 levels and significantly higher IgG reactivity against IL‐6 (Figure 2, Table 1). Clinically, this group was significantly younger than the combined set of patients of the other groups and tended toward higher SNN scores, suggestive of a more widespread disorder (Table 1).

Group C comprised 8 patients, with 2 exhibiting proximity to group A (Figure 1). This group was characterized by significantly higher IL‐17 levels and elevated IgG reactivity against IFN‐γ (Figures 2 and 3, Table 1). Clinically, they tended to have a lower proportion of cases with SNN scores exceeding 10 (Table 1).

Group D, although displaying relative variability among the samples, remained distinctly separate from the others (Figure 1). Composed of only 3 patients, this group was the youngest among (28.0 ± 2.9 years) and exhibited remarkably high level of TNF‐α, IL‐1β, and IL‐6 (Figure 2, Table 1).

### 3.2 Relationship Between Cytokine Profile and SNN Score

When comparing patients with SNN scores above 10 with those below 10, several cytokine distinctions emerged. Patients with higher SNN scores exhibited elevated TNF‐α levels (165.3 ± 282.1 vs. 134.3 ± 185.6 pg/mL, *p* = 0.016, Mann–Whitney test), reduced IFN‐γ levels (10.2 ± 26.7 vs. 20.4 ± 34.8 pg/mL, *p* = 0.01, Mann–Whitney test), and lower anti‐IFN‐γ immunoreactivity (OD 0.50 ± 0.49 vs. 0.87 ± 0.62, *p* = 0.001, Mann–Whitney test).

Sub‐analyses confined to group A, constituting the majority of patients, yielded similar findings. Individuals with SNN scores exceeding 10 displayed higher TNF‐α levels (132 ± 78.8 pg/mL vs. 101.0 ± 86.4 pg/mL, *p* = 0.014, Mann–Whitney test), lower IFN‐γ levels (8.2 ± 23.6 vs. 19.2 ± 34.5 pg/mL, *p* = 0.018, Mann–Whitney test), and diminished anti‐IFN‐γ immunoreactivity (OD 0.50 ± 0.40 vs. 0.85 ± 0.69, *p* = 0.015, Mann–Whitney test).

### 3.3 Correlation Between Cytokine Profile and mRS Score

Among the 73 patients with available baseline mRS scores, no correlations were observed between mRS score and the levels of any cytokine or their respective immunoreactivities (data not shown).

### 3.4 Relationship Between Cytokine Profile and Response to Immunomodulatory Treatment

Forty‐seven patients receiving immunomodulatory treatment and evaluated with the mRS both before and after treatment were included in this analysis. Eleven individuals were classified as responders due to demonstrating at least a 1‐point improvement on the mRS [7]. Responder status was not associated with differences in cytokine levels or anti‐IL‐6 or anti‐INF‐γ immunoreactivity (data not shown).

### 3.5 Relationship Between Cytokine Profile and Associated Immune Context

To assess potential associations between cytokine profiles and the presence of anti‐AGO antibodies, anti‐FGFR3 antibodies, or an associated autoimmune disease (excluding the aforementioned antibodies), cytokine levels were compared between these groups and the remainder of the studied population lacking identifiable autoimmune contexts. No significant differences in cytokine profiles were observed among the three groups. Additionally, Spearman correlation analyses revealed no relationship between the levels of anti‐AGO or anti‐FGFR3 antibodies (expressed as *z*‐scores) and the levels of any cytokine (data not shown).

### 3.6 Correlations Among Blood Cytokines and Anti‐Cytokines Immunoreactivity

To investigate potential correlations among circulating cytokine levels and with anti‐IL‐6 or anti‐IFN‐γ immunoreactivities, Spearman's rho coefficient was calculated for the entire cohort and each subgroup. Several weak yet statistically significant correlations emerged.

Within the total SNN cohort, TNF‐α levels correlated with those of IFN α‐2 (Rho = 0.25, *p* = 0.009) and IL‐6 (Rho = 0.32, *p* = 0.001). Levels on IFN α‐2 also correlated with those of IFN‐γ (Rho = 0.24, *p* = 0.013; Table S1). No correlations were observed between cytokine levels (IL‐6 and IFN‐γ) and corresponding autoantibody reactivities. However, anti‐IFN‐γ immunoreactivity levels correlated with those IL‐17 (Rho = 0.24, *p* = 0.019).

Within group A, IFN α‐2 levels correlated with those of IL‐1β (Rho = 0.20, *p* = 0.044), IL‐17 (Rho = 0.31, *p* = 0.002), IFN‐γ (Rho = 0.24, *p* = 0.017), and TNF‐α levels (Rho = 0.21, *p* = 0.039). Additionally, TNF‐α levels correlated with those of IL‐6 (Rho = 0.24, *p* = 0.002), anti‐IFN‐γ immunoreactivity levels correlated with those IL‐17 (Rho = 0.22, *p* = 0.046).

In group B, a correlation existed between IL‐17 and IL‐1β levels (Rho = 0.76, *p* = 0.046).

No significant correlations were observed within groups C and D.

## 4 Discussion

Our study explored profiles of selected pro‐inflammatory cytokines and IgG ACAAs in patients experiencing SNN with suspected immune‐mediated pathology. Criteria for inclusion included: (a) presence of anti‐AGO or FGFR3 antibodies, (b) coexistent autoimmune disorders (particularly Sjögren syndrome), or (c) clinical presentation indicative of an immune‐driven process.

We identified four distinct subgroups based on unique patterns of pro‐inflammatory cytokines (IL‐1β, IL‐6, IL‐17, TNF‐α, IFN α‐2, and IFN‐γ). This heterogeneity suggests a multifaceted pathophysiology underlying immune‐mediated SNN, underscoring the need for tailored therapeutic approaches. The predominant subgroup comprises 84% of cases and was characterized by comparatively low circulating cytokine levels and advanced age compared with the remaining three groups.

The pro‐inflammatory cytokines analyzed in this study exhibit a wide array of actions influencing both inflammation and the wider immune response [13].

IL‐1 plays a crucial role in initiating the inflammatory cascade by activating macrophages, B, T, and NK cells, along with promoting the expression of adhesion molecules on endothelial cells, thereby facilitating the recruitment of immune cells to the site of inflammation [10, 13, 20, 21].

IL‐6 exerts a broad spectrum of effects, contributing to the acute phase response, regulating the balance between Th1 and Th2 lymphocytes, and playing a vital role in antibody production and Th‐17 cell differentiation [22, 23, 24].

TNF‐α acts as a potent activator of cells involved in the innate immunity, including neutrophils, macrophages, and NK cells. Interestingly, depending on the context, TNF‐α can activate pathways leading to either pro‐inflammatory or anti‐inflammatory outcomes [13, 25, 26].

Finally, IL‐17 drives T cell proliferation and promotes the production of chemokines associated with Th17 cells, thereby contributing to the chronicity of the inflammatory response, particularly in concert with TNF‐α [10, 27, 28].

Interferons play a crucial role in both the innate and adaptative immunity, with production typically triggered by viral infections. A primary function of INF‐α is the stimulation of antigen‐presenting cells. It also contributes to B‐cell activation and modulates T‐cell activity [29, 30, 31]. IFN‐γ, primarily released by Th1 cells, is a potent activator of macrophages and induces the expression of type I and II MHC molecules, which are fundamental to both the innate and adaptative immune responses [13, 32]. Over recent years, interferons have been recognized as key factors and potential therapeutic targets in various autoimmune diseases, including lupus [33], Sjögren syndrome [30], and rheumatic arthritis [34]. Elevated blood levels of these cytokines are frequently observed during active phases of these diseases [13].

However, it is important to note that local production of interferons and their effects on tissue lesions may not consistently correlate with systemic blood levels.

Differences observed between the subgroups were not attributable to the delay between the neuropathy onset and blood sampling, nor to the disease course, as neither factor differed significantly between the groups. It is well established that blood cytokine levels, particularly IL‐6, tend to increase with age and are associated with senescence and the development of autoimmune diseases [35].

This observation initially appears paradoxical, as patients in group A, despite their older age, exhibited comparatively lower cytokine levels. Nevertheless, their absolute levels remained elevated. For instance, the mean IL‐6 level in group A was 14.0 + 21.3 pg/mL, whereas a meta‐analysis estimated the 95% confidence interval for healthy adults to be 4.63–5.74 [36]. Similarly, their mean TNF‐α level was 123.0 + 81.2 pg/mL, exceeding typical levels of below 80 pg/mL observed in healthy controls.

Though age likely contributed to the elevated cytokine levels observed, it is unlikely to be the sole explanatory factor. High cytokine levels likely also reflect the underlying disease process. Supporting this notion, in group A, patients with more extensive disease, as indicated by higher SNN scores (> 10), exhibited higher TNF‐α levels and lower IFN‐γ levels compared to those with lower SNN scores (≤ 10). This suggests a dynamic interplay between these cytokines and disease progression. TNF‐α is primarily known for activating innate immunity and triggering inflammation, whereas IFN‐γ plays a crucial role in the Th1 response [25, 37].

Younger patients in group B, who distinctively diverged from other groups, exhibited high circulating levels of IFN α‐2. Produced in response to both exogenous stimuli (such as viral pathogens) and endogenous stimuli (such as self‐nucleic acids), IFN α‐2 is generated by various cell types, with plasmacytoid dendritic cells being among the most potent producers [10, 13]. IFN α‐2 regulates autoimmune processes and carries out its activity on multiple immune pathways, including the differentiation and activation of CD4+ T‐cells and B‐cells [12].

Clinically, these patients tended towards higher SNN scores, indicating more widespread involvement of DRG. Though the small number of patients in this group resulted in only marginally significant findings, these trends likely reflect a genuine phenomenon. Crucially, the cytokine profile in this group differed from that seen in group A patients with SNN scores > 10, suggesting distinct underlying mechanisms in these two groups.

Group C, consisting of eight patients, demonstrated elevated levels of IL‐17. Primarily produced by CD4+ Th17 lymphocytes, IL‐17 is also secreted by other cell types, including CD8+ cytotoxic T cells, NK cells, dendritic cells, macrophages, and neutrophils [33]. IL‐17 participates in a complex cytokine network that amplifies inflammatory responses and contributes to tissue damage. It also stimulates the production of other pro‐inflammatory cytokines, such as IL‐1 and IL‐6 [33]. Clinically, these patients tended to have a lower frequency of SNN scores > 10, indicating a more localized involvement predominantly affecting the lower limbs.

Finally, group D, comprising only three younger patients, presented a unique profile characterized by exceptionally high levels of TNF‐α, IL‐1β, and IL‐6, pointing towards a robust ongoing pro‐inflammatory state. However, the limited sample size makes it challenging to delve deeper into characterizing this group.

Irrespective of the four distinct groups, correlational patterns emerged across several cytokines. Notably, levels of strongly pro‐inflammatory cytokines TNF‐α and IL‐6 correlated with each other. Within subgroups, strong correlations emerged between IL‐17 and IL‐1β in group B. IL‐1β, produced by monocytes and polynuclear cells, has been shown to play a pathogenic role by inducing IL‐17 production by various T‐cell subsets [38].

One noteworthy finding of this study is that the presence of anti‐AGO or anti‐FGFR3 antibodies, which clinically delineate subgroups of SNN or associate with autoimmune conditions like Sjögren's syndrome, did not correlate with any distinctive cytokine profile. This held true even for patients with the highest levels of seropositivity (data not shown). While the smaller sizes of groups B, C, and D might have limited our ability to detect differences, it is also plausible that the restricted set of cytokines examined in this study contributed to this outcome. Notably, we did not include cytokines such as IL‐7, IL‐21, IL‐4, and IL‐10, which are crucial for B‐cell development and activation [39]. Further exploring these cytokines might reveal connections currently obscured.

Recent studies have highlighted potential importance of anti‐cytokine antibodies in modulating immune responses during both infectious and autoimmune diseases. Our study uncovered a link between the presence of anti‐IL‐6 and anti‐IFN‐γ immunoreactivity and specific cytokine profiles, potentially influenced by the age of the patients. Specifically, patients in group B, one of the two younger patient groups, exhibited the highest levels of anti‐IL‐6 immunoreactivity. Patients in group C displayed high levels of anti‐IFN‐γ immunoreactivity. Further investigations are necessary to determine whether these antibodies are merely passive products of cytokine secretion or whether they actively participate in shaping the immune response.

## 5 Conclusion

Analyzing blood levels of pro‐inflammatory cytokines in patients with immune‐mediated SNN unveiled distinct cytokine profiles. These profiles correlated with patient age, the extent of DRG involvement, and the presence of anti‐cytokine immunoreactivity. These findings suggest that personalized treatment strategies targeting specific cytokines might prove beneficial for strategically selected patient subgroups.

## Conflicts of Interest

C.P.M. accepted covered travel and lodging from Argenx to present at a conference in Paris, not related to this project. C.P.M. also submitted a patent application for the application of AGO antibodies as biomarkers for autoimmune neurologic diseases. J.‐P.C. declares that he received or has received fees for lectures, consulting, writing of articles, or training courses from Akcea, Alexion, Alnylam, Argenx, Biogen, Bristol Myers Squibb, CSL‐Behring, Genzyme, Grifols, GSK, LFB, Merck, Novartis, Pfizer, Pharmalliance, Teva, UCB Pharma, Editions Scientifiques L&C, Edimark, Expression Santé, Natus, Scien, SNF‐Floerger and that he has submitted holds a patent on anti‐FGFR3 antibodies and submitted a patent application for AGO antibodies as biomarkers for autoimmune neurologic diseases. J.‐C.A. holds a patent on anti‐FGFR3 antibodies and has submitted a patent application for the application of AGO antibodies as biomarkers for autoimmune neurologic diseases. He received fees from CSL Behring for scientific counselling. The other authors declare no conflicts of interest.

## Supporting information


**Table S1.** Analysis of correlations between levels of cytokines among each other and anti‐cytokine reactivities.

## Data Availability

The data that support the findings of this study are available from the corresponding author upon reasonable request.
